# Implementation of a community-based LC-UV drug checking service: promising preliminary findings on feasibility and validity

**DOI:** 10.1186/s12954-024-01098-4

**Published:** 2024-10-18

**Authors:** Nicolas Fabresse, Eurydice Papias, Alma Heckenroth, Victor Martin, Daniel Allemann, Perrine Roux

**Affiliations:** 1 Laboratory of Pharmacokinetics and Toxicology, La Timone University Hospital, 264 rue Saint Pierre, Marseille Cedex 5, 13385 France; 2 Aix Marseille University, INSERM, IRD, SESSTIM, Economic and Social Sciences of Health and Medical Information Processing, Marseille, France; 3 BUS 31/32 Association, 129 Avenue de Toulon, Marseille, 13005 France; 4 Aix Marseille Univ, IRD, LPED, Marseille, France; 5 Health, Social and Integration Directorate of the Canton of Bern, Bern, Switzerland

**Keywords:** Harm reduction, Drug checking, LC-UV, LC-HRMS, Cross-validation, Psychoactive substances

## Abstract

**Background:**

The increasing diversity of psychoactive substances on the unregulated drug market poses significant health, psychological, and social risks to people who use drugs (PWUD). To address these risks, various harm reduction (HR) policies have been implemented, including drug checking services (DCS). Many analytical methods are used for DCS. While qualitative methods (e.g., thin layer chromatography, spectroscopy) are easier to implement, they are not as accurate as quantitative methods (e.g., LC-UV, LC-MS). Some HR programmes have implemented high-performance liquid chromatography coupled with UV detection (LC-UV). This article presents the cross-validation of this quantitative method with a reference liquid chromatography coupled with high resolution mass spectrometry (LC-HRMS) method.

**Methods:**

Drug samples were provided by PWUD to a DCS called DrugLab in Marseille, France. The samples were weighed and prepared through dissolution in methanol, followed by ultrasonic bathing. Samples were analysed onsite using LC-UV analysis. They were then subsequently analysed with the reference LC-HRMS method. The LC-UV instrument in DrugLab was calibrated after being purchased; analysis of standard solutions was routinely performed once a month and after maintenance operations. For the LC-HRMS instrument, calibration and quality control procedures followed European Medicines Agency (EMA) guidelines. Statistical analyses were conducted including Spearman correlation tests using IBM^®^ SPSS^®^ Statistics version 20.

**Results:**

A total of 102 samples representing different product classes and cutting agents were cross-validated. Differences between both analyses methods for each molecule analysed were ≤ 20%, with significant correlations between both methods’ results for most substances. Notably, LC-HRMS provided lower concentration values for cocaine and acetaminophen, whereas it provided higher values for other substances. Correlations were significant for cocaine, ketamine, MDMA, heroin, amphetamine, caffeine, acetaminophen, and levamisole.

**Conclusions:**

This study demonstrates that the results provided by DrugLab were accurate and reliable, making LC-UV an adaptable, stable, and suitable analytical method for simple matrices like drugs in a DCS context. However, this cross validation does not guarantee accuracy over time. A proficiency test project in HR laboratories across France is currently under development in order to address potential drifts in LC-UV accuracy.

## Introduction

Given the increasing diversity of psychoactive substances available on the unregulated drug market and the associated health, psychological, and social risks to people who use drugs (PWUD), many countries have developed harm reduction (HR) policies [1]. In France, various HR programmes have been developed since the 1980s by the *Médecins du Monde* (MdM) association in a context of increasing injection drug use associated with the HIV epidemic. Different HR tools have been implemented in the country, including needle exchange programs, the provision of sterile injection equipment, access to opioid agonist therapy (methadone and buprenorphine), and more recently, drug consumption rooms [2] and drug-checking services (DCS).

DCS were developed as a response to the risk of potentially lethal adulteration of psychoactive substances by drug dealers who intentionally mix several substances - psychoactive or not - during the drug manufacturing process in order to increase quantity and therefore profit [3–5]. These services provide a chemical analysis of products from the unregulated market. Specifically, they identify the substances in a drug sample, provide the individual PWUD with this information, and suggest ways to reduce associated risks.

Drug checking is widely used in different contexts and by many different PWUD populations (e.g. partygoers, people who inject drugs). In France, the first DCS were implemented in 1997, initially in mobile off-site units in party settings, such as raves and music festivals, using testing kits. Gradually, DCS expanded beyond these events, particularly into HR programmes and using with laboratory-based analytical methods.

Several qualitative and quantitative analytical techniques are used for drug checking. Each has advantages and limitations, regarding speed, cost, complexity of use, and especially reliability. With regard to qualitative techniques (e.g., thin-layer chromatography (TLC), spectroscopy-based techniques), advantages include the fact that they are easy to implement in HR programmes. Furthermore, although they require trained persons to interpret results, these persons do not need to be highly qualified. In terms of limitations, TLC only identifies ‘classic’ substances in a drug sample (e.g., cocaine, LSD, MDMA). It cannot identify unusual or volatile substances (e.g., poppers, solvents, nitrous oxide). Additionally, compounds present in low concentrations may go undetected [6]. With regard to spectroscopy-based techniques (e.g., infrared, Fourier transform infrared, Raman), just as is the case for TLC, the reliability of analyses is limited for mixtures and compounds in low concentrations [6–9].

In terms of the advantages of quantitative drug checking techniques, nuclear magnetic resonance (NMR) provides the chemical structure of the molecules analysed, and thus the identification of substances that have never been detected before. If purity is sufficient, substances can also be quantified. However, it requires highly qualified personnel and pure samples, making it impractical for DCS in community-based settings. Liquid chromatography (LC) coupled with high resolution mass spectrometry (LC-HRMS) provides both the sample composition and quantified results. Specifically, by measuring the exact mass of compounds in a sample, LC-HRMS can determine the chemical formula of molecules, thereby providing reliable molecule identification and concentration data. However, just like NMR, it also requires highly qualified staff and specific equipment making it impractical for community-based settings [6, 10]. High-performance liquid chromatography coupled with UV detection (LC-UV) is another quantitative method and is the foundation for the work described here.

In 2020, the PWUD community-based association Bus 31/32, based in Marseille, France, developed a quantitative-based DCS called DrugLab as part of its HR programme. Specifically, DrugLab acquired a high-performance LC-UV system to perform analyses and to screen for a wide range of psychoactive compounds. Highly qualified staff were hired, and a laboratory space and other necessary equipment were set up. Certified reference material (CRM) was obtained for the main narcotic substances consumed by PWUD. The LC-UV method to be used by DrugLab was adapted from a method developed by the Bern Cantonal Pharmaceutical Service, which is the competent authority for therapeutic products and narcotics in the canton of Bern, Switzerland [11]. This analytical method was adapted and implemented by DrugLab in such a way as to ensure that substances could be quantified quickly and accurately.

This paper presents the cross-validation of the quantitative analysis used in DrugLab with the liquid LC-HRMS analytical system used in the Pharmacokinetics and Toxicology Laboratory at the University Hospital ‘La Timone’ in Marseille, as well as the entire process involved in the implementation and the maintenance of the adapted analytical method used by DrugLab.

## Materials and methods

### DrugLab description and LC-UV method

#### A drug checking service as part of a PWUD community association’s HR program

Samples are collected in an anonymous and non-judgmental context either directly at the Druglab premises or in party settings using an off-site mobile unit. When providing their product sample for analysis, PWUD also participate in a face-to-face conversation that focuses on the product, the mode of consumption, HR tools in general, the potential interactions between the product and other drugs and/or medications and their associated risks, as well as potentially referring the person to a specific healthcare pathway. The aim of this brief complementary intervention is to foster PWUD’s self-management of their drug use by encouraging greater awareness of the substances they consume.

DrugLab analyses samples three or four days a week, depending on the number of samples which have been collected for examination in the previous days. On three designated half-days a week, PWUD can bring their samples for real-time, immediate analysis. Meetings with PWUD and external association-based partners working with PWUD are also organised at the DrugLab premises. Moreover, DrugLab has a dedicated website for publicly sharing analysis results and disseminating the same information on psychoactive substances provided during the complementary intervention described above (https://druglab.fr/). The DrugLab analysis team comprises a pharmacist, a laboratory engineer, an analytical chemistry undergraduate trainee, and a project manager.

In the following paragraphs, we describe the methods used at the DrugLab and at the Pharmacokinetics and Toxicology Laboratory for the cross-validation analysis described in this paper.

### DrugLab’s LC-UV method

#### Reagents

Purified water and methanol $\:\ge\:$ 99.8% were obtained from Thermo Fisher Scientific (Fisher Chemical) (Hampton, New Hampshire, United States). Acetonitrile, phosphoric acid 85% were purchased from Sordalab (Etampes, France). Hexylamine 99% was obtained from Acros Organics (Geel, Belgium). Powder reagents containing 10 mg of cocaine, ketamine, MDMA, amphetamine, heroin, and cutting agents (caffeine, acetaminophen, levamisole) were supplied by LGC Standard (Molsheim, France).

#### Sample preparation

Upon receipt of samples, photographs were taken and a macroscopic examination (colour, tablet dimensions, powder appearance) was conducted. Sample preparation differed according to the form of the product to be analysed. For powders, between 9 and 15 mg were weighed and dissolved in a 25 mL volumetric flask, completed with methanol. An exception was made for MDMA crystal samples, for which the amount should not exceed 10 mg per 25 mL. The flask was then placed in an ultrasonic bath for 3 min to homogenize the solution. Finally, the sample solution was filtered into a vial using a PES syringe filter Branchia 0.22 μm (Labbox, Rungis, France). Two µL were injected into the chromatographic system.

#### Analysis conditions

##### Liquid chromatography

Liquid chromatography was performed using a Jasco PU-4185 Binary unit (Lisses, France). The compounds were eluted on a ProntoSIL Spheribond 80-3-ODS1 C18 column (125 × 4,0 mm; 3 μm; Bischoff Chromatography, Leonberg, Germany).

Mobile phase A comprised 8.5 g of phosphoric acid, 560 µL of hexylamine and completion with purified water for a total volume of 1000 mL. Then, 1 mL of acetonitrile was added to stabilize the solution. Mobile phase B was constituted of 4.25 g of phosphoric acid, 280 µL of hexylamine, 45.75 g of purified water, and 351 g of acetonitrile.

- Method 1: cocaine, heroin, and cutting agents.

The flow rate was held at 1.5 mL/min. The gradient was as follows: 7% B for 1.50 min, linear gradient to 56% B for 4.50 min and held for 3 min, linear gradient to 7% B for 0.35 min. The column was then equilibrated to initial conditions for 2.25 min. Total run time was 12 min.

- Method 2: MDMA, ketamine, and amphetamine.

The flow rate was held at 1 mL/min. The gradient was as follows: 5% B for 1 min, linear gradient to 56% B for 5 min and held for 3 min, linear gradient to 5% B for 0.35 min. The column was then equilibrated to initial conditions for 2.25 min. Total run time was 12 min.

##### UV detection

UV detection analysis was performed using a Jasco AS-4050 detector (Lisses, France). UV detection was conducted in the 190–400 nm range at 198 nm.

#### Calibration and quality control procedures

The LC-UV system was calibrated after it was purchased. Calibration standards at 950 µg/mL were prepared from drug standards for each molecule with methanol in order to obtain a calibration range comprising 6 levels (95 µg/mL, 190 µg/mL, 285 µg/mL, 380 µg/mL, 475 µg/mL, and 570 µg/mL). The limit of detection (LOD) was experimentally estimated to be 1 µg/mL with a 2 µL injection. Routine calibration is performed once a month and after maintenance operations. Products containing substances not included in the DrugLab library are sent to the Pharmacokinetics and Toxicology Laboratory in Marseille (see above) for analysis with LC-HRMS, or to an NMR platform in Université de Bretagne Occidentale, located in Brest, France for identification and quantification. Molecules identified with LC-HRMS and/or the NMR platform are then added to the DrugLab library (spectra and retention time) for future identification. Samples of new psychoactive substances (NPS) analysed by NMR are also used to produce ‘in-house’ standards. As of September 2024, 16 samples had been analysed by NMR for identification and exploration of composition purposes. This collaboration between DrugLab and the Université de Bretagne Occidentale will continue into the future to ensure quick responses in an ever-changing drug market.

### The cross-validation reference method LC-HRMS at the Pharmacokinetics and Toxicology Laboratory, ‘La Timone’ Hospital

#### Reagents

Water purity was 18.2 mΩ/cm (Millipore, France). Acetonitrile, formic acid 98% and methanol were obtained from VWR International (Radnor, Pennsylvania, United States). Ammonium formate was purchased from Sigma Aldrich (Munich, Germany). Drug standards at 1 mg/mL of cocaine, ketamine, heroin, amphetamine and acetaminophen are purchased from Euromedex (Souffelweyersheim, France), those of MDMA, caffeine and levamisole are distributed by LGC Standard (Molsheim, France).

#### Sample preparation

Upon receipt of samples, a macroscopic examination (colour, tablet dimensions, powder appearance) was conducted. Ten milligrams of powder or ground tablet were weighed and placed into a 10 mL volumetric flask, which was then completed with methanol. The solution was placed in an ultrasonic bath for 10 min to dissolve the sample. It was then transferred into a 15 mL vial and stored at + 4 °C until the day of analysis. On the day of analysis, two dilutions of the samples were prepared with phase A in a hemolysis tube. Mobile phase A comprised purified water, formic acid 0.1%, and ammonium formate 2mM. A 1:100 dilution was prepared by mixing 10 µL of the sample with 990 µL of phase A. A 1:1000 dilution was achieved through a series of three sequential 1:10 dilutions, each made by combining 100 µL of the sample with 900 µL of phase A. Twenty-five µL of internal standard solution was added to all the samples. One µL was injected into the chromatographic system.

#### Analysis conditions

##### Liquid chromatography

Liquid chromatography was performed using a Vanquish system (Thermo Fisher Scientific, Les Ulis, France). The compounds were eluted on a Luna^®^ Omega Polar C18 column (100Ä, 100 × 2.1 mm; 1.6 μm; Phenomenex, Le Pecq, France). Mobile phase B comprised methanol. The flow rate was held at 400 µL/min. The gradient was as follows: 0% B for 1 min, linear gradient to 35% B for 3 min, linear gradient to 45% B for 1 min, linear gradient to 50% B for 1 min and held for 1 min, linear gradient to 80% B for 2 min, linear gradient to 100% B for 2 min and held for 1 min. The column was then equilibrated in initial conditions for 2 min. Total run time was 14 min.

##### High resolution mass spectrometry

Mass spectrometry analysis was performed using an Exploris 120 mass spectrometer (Thermo Fisher Scientific, Les Ulis, France) coupled with a heated electrospray ionization probe (HESI). Ion source settings were as follows: sheath gas = 60 arbitrary unit (AU), auxiliary gas = 10 AU, sweep gas = 0 AU, vaporization temperature = 320 °C, ion spray voltage = 3.5 kV, S-Lens = 70 eV. Analysis was performed in the data dependent analysis (DDA) mode. A full scan analysis was performed with a mass resolution of 60,000 FWHM within the 125–650 m/z mass range, followed by four cycles of DDA with a mass resolution of 16,000 FWHM. The intensity threshold for the DDA was set at 5.0 e^5^. The mass window for precursor ion selection was fixed at 1 Da. Raw data were acquired using Xcalibur software (v.4.0, Thermo Fisher Scientific, USA).

#### Calibration and quality control procedures

Linearity was validated with a calibration range comprising 6 levels (10 ng/mL, 25 ng/mL, 50 ng/mL, 100 ng/mL, 500 ng/mL, and 1000 ng/mL). The analyte / internal standard chromatographic peak area ratios were used for concentration calculations. Three quality controls (QCs) were analysed before and after the sample series (30 ng/mL, 300 ng/mL, and 750 ng/mL). The LOD was experimentally estimated to be 1 ng/mL.

The method’s technical validation criteria were as follows:

- Correlation coefficient > 0.99 for the calibration curve,
- Bias < 15% for calibration points, and < 20% for the limit of quantification, Bias < 15% for at least two-thirds of internal quality controls.
- A calibration range was established for each series of sample analyses. The method was validated according to European Medicines Agency (EMA) guidelines.

### Cross-validation

A cross-validation analysis was performed to validate the reliability of the results produced by DrugLab. Powder samples representing the different substance classes were transmitted directly (i.e., hand to hand) by DrugLab staff to the Pharmacokinetics and Toxicology laboratory. Both methods (i.e., LC-UV at DrugLab and LC-HRMS at the hospital laboratory) were used to analyse the samples. The results obtained were compared to determine the significance of the correlation and the bias between both methods. Correlation was assessed using the Spearman test. Statistical analyses were conducted using the SPSS^®^ software (v.20, IBM^®^, USA). The bias was calculated for all the products analysed, using LC-HRMS as the reference method. A bias ≤ 20% was considered acceptable, in accordance with the hospital laboratory’s analytical validation criteria.

To illustrate the value of collaboration with an institutional laboratory and the potential for continuously adding to the list of substances in the DrugLab’s LC-UV spectral library, we present some of the samples for which the LC-UV method did not identify a substance, but which was identified by the LC-HRMS method (see below).

## Results

The cross-validation analysis was performed on a total of 102 powder-based samples representing different product classes and cutting agents. Specifically, there were 24 samples of cocaine, 21 samples of ketamine, 20 samples of MDMA, 19 samples of heroin and 18 samples of amphetamine. Additionnaly, 3 cutting agents were quantified: caffeine in 19 samples of heroin, acetaminophen in 16 samples of heroin, and levamisole in 9 samples of cocaine.

Table 1 presents the medians of the concentrations measured by LC-HRMS and LC-UV for each molecule analysed and the associated biases. The calculated biases for all molecules analysed were $\:\le\:$ 20%. For the cocaine and acetaminophen samples, a negative bias was found, indicating that on average, the LC-HRMS method provided a lower concentration value compared to the LC-UV method. All other samples provided a positive bias, indicating that LC-HRMS gave higher concentration values.

**Table 1:** Cross-validation of the analysis results of psychoactive substances and their cutting agents by LC-HRMS and LC-UV

				Bias (%)		Spearman correlation test	
Molecule	*n*	Median LC-HRMS (%)	Median LC-UV (%)	Median (min-max)	Standard deviation	Correlation coefficient	*p*-value
Cocaine	24	57	74	-13 (-19–10)	7.65	0.966	< 0.001
Ketamine	21	98	96	5 (-14–14)	6.79	0.691	0.001
MDMA	20	37	30	5 (3–18)	5.55	0.908	< 0.001
Heroin	19	13	10	4 (-13–12)	6.33	0.944	< 0.001
Amphetamine	18	53	38	3 (-7–16)	6.17	0.986	< 0.001
Caffeine	19	28	23	4 (-19–17)	6.81	0.712	0.001
Acetaminophen	16	52	58	-7 (-14–11)	6.26	0.795	< 0.001
Levamisole	9	13	12	1 (-15–14)	8	0.661	0.05

The correlation between the two methods was assessed using a Spearman correlation test. Results obtained by LC-HRMS and LC-UV were significantly correlated for cocaine (rs = 0.97; *p* < 0.001), ketamine (rs = 0.69; *p* = 0.001), MDMA (rs = 0.91; *p* < 0.001), heroin (rs = 0.94; *p* < 0.001), amphetamine (rs = 0.99; *p* < 0.001), caffeine (rs = 0.71; *p* = 0.001), acetaminophen (rs = 0.79; *p* < 0.001), and levamisole (rs = 0.66 ; *p* = 0.05).

**Fig. 1:**
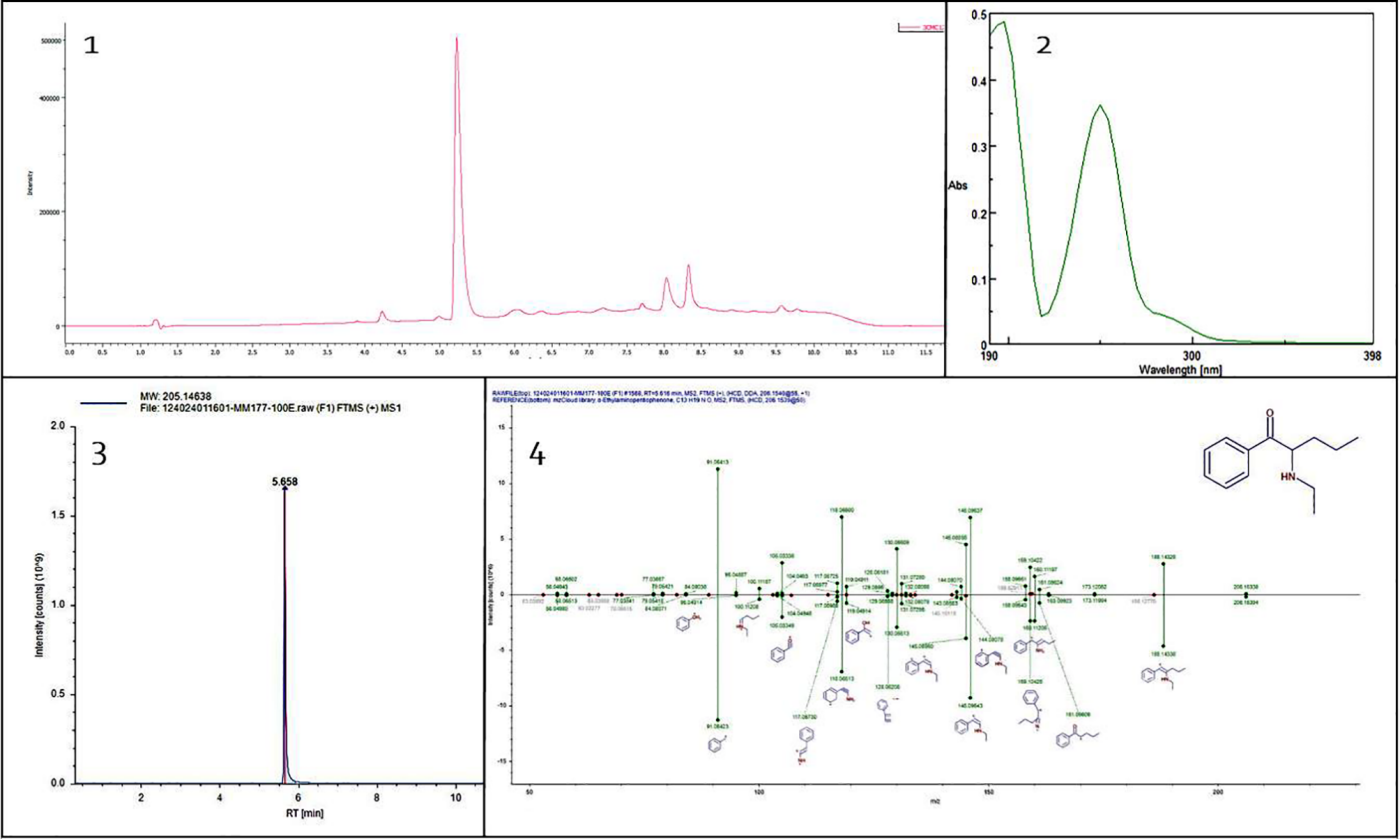
Results of NEP analysis using both methods (1) LC-UV chromatogram, (2) UV spectra (190–400 nm), (3) LC-HRMS chromatogram (extracted ion chromatogram [EIC] of m/z = 206.1539), (4) experimental (top) and reference (bottom) high resolution mass spectra for NEP

Twelve products analysed by the DrugLab detected compounds with chromatographic peaks associated with characteristic absorbance spectra which however were not identifiable by LC-UV as they were not listed in the system’s spectral database. These products were analysed with LC-HRMS and the composition was successfully reported. Nine matched the substance identification provided by the PWUD (i.e., they matched the substance which the PWUD believed they had bought) as follows: 2,5-Dimethoxy-4-ethylphenethylamine (2 C-E), 2,5-Dimethoxy-4-methyl-phenethylamine (2 C-D), 1-valeryl-D-lysergic acid diethylamide (1v-LSD), 1-propanoyl-lysergic acid diethylamide (1p-LSD), 3-Methyl-PCP (3-MePCP), 3-fluoromethamphetamine (3-FMA), 4-hydroxy-N-methyl-N-ethyltryptamine (4-HO-MET), 6-(2-aminopropyl)benzofuran (6-APB), methoxpropamine (MTXr). For three products, users did not know the composition: N-ethylpentedrone (NEP), Dipentylone, 3,4-dimethylmethcathinone (3,4-DMMC). Accordingly, the LC-UV spectral library was successfully expanded for the future identification of these three molecules. Figure 1 presents the chromatograms and spectra obtained with both methods for NEP.

## Discussion

Our LC-UV LC-HRMS cross-validation analysis highlighted that LC-UV is a reliable method for DCS in the context of HR programs for PWUD. More specifically, for the main substances used by PWUD (cocaine, ketamine, MDMA, heroin, amphetamine) and for associated cutting agents (caffeine, acetaminophen, and levamisole), there was no significant bias (≤ 20%) between the results obtained with LC-UV and those obtained with a validated LC-HRMS method. Moreover, good correlation was found between both methods.

**Fig. 2:**
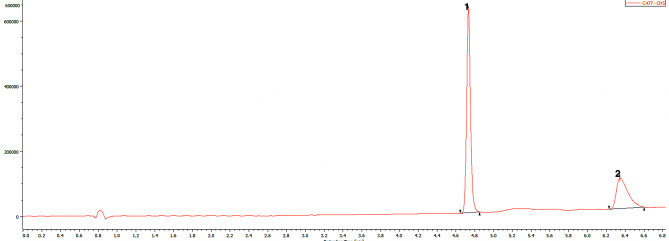
LC-UV chromatogram (198 nm) of a mixed sample containing 54% phenacetine (peak 1) and 46% cocaine (peak 2)

The most widely used DCS use non-separation-based qualitative methods which have been described in detail [6, 9, 12–14]. However, in recent years, some HR programmes have chosen to equip themselves with LC-UV systems to provide quantitative analyses [15, 16]. LC-UV combines a separation-based method (i.e., liquid chromatography) with a detection system based on ultraviolet light absorption. Unlike non-separation-based methods, this enables specific quantitative analyses to be performed. More specifically, the spectra acquired are specific to each separated molecule (Fig. 2), rather than reflecting a possible mixture of several molecules This is essential for the analysis of cocaine and heroin samples, as in the vast majority of cases, these products are mixed with other substances, making them difficult to analyse using IR. Moreover, by measuring the absorbance intensity at a given ultraviolet wavelength with LC-UV, we can deduce the concentration present in the sample, and produce a quantitative result, unlike infrared spectroscopy or TLC.

LC-HRMS systems are sophisticated and complex instruments, requiring daily calibration to guarantee accurate results. In contrast, after initial calibration following its acquisition, DrugLab’s LC-UV system only requires calibration on a monthly basis and after maintenance operations to ensure accuracy. During the period of the cross-validation analysis described here, the LC-UV results were accurate and stable. This difference in calibration requirements between these two methods can be explained by the robustness and stability of the relationship between signal and concentration with UV detection, which is not the case for mass spectrometry. Therefore, for simple matrix analysis (e.g., psychoactive drugs but not biological samples), we recommend that HR laboratories use LC-UV, especially given that the method can be adapted to allow new molecules to be included in a DCS in a context of ever-changing trends in the unregulated drug market.

LC-UV-based DCS are a new HR service providing PWUD the opportunity to have their drug samples analysed quantitatively, and to receive more accurate and complete information about the drugs they use. Quantification is very important in drug checking, as the substances analysed may be present in highly variable concentrations. For example, the heroin samples analysed as part of our cross-validation process ranged in concentration from 3 to 60%. Typical levels are around 10%. If a user consumes a product with 60% heroin in the same way as he/she would a product with 10% heroin, the risk of overdose is high. In this context, the messages delivered by DCS following analysis enable PWUD to adapt their doses and limit risks. Heroin and cocaine, which account for the majority of products analysed by DrugLab, are almost systematically adulterated (e.g., with up to 90% cutting agents). The main cutting agents used for heroin (acetaminophen, caffeine) and cocaine (levamisole) can also be quantified using the LC-UV method, to ensure they present no risk.

For some of the 102 samples in our validation analyses, differences of up to 20% were observed between the two methods. These differences can be explained by the different analytical conditions employed in each case (laboratory, personnel, equipment, and dilutions). While a 20% bias may seem high, it nonetheless meets DCS objectives, which are to highlight the presence of the supposedly present substance (i.e., the substance the PWUD believes that he/she had purchased) at a concentration close to the expected one, the presence of another substance, or the absence of the supposedly present substance.

Some molecules are particularly potent pharmacologically (e.g., nitazenes, fentanyloids); for these molecules, a small change in dose can lead to a risk of overdose. None of these compounds are expected to be found in the samples which DrugLab routinely analyses. The fact that we found them indicates product adulteration which in turn represents a major risk of intoxication. When these types of molecules are found, the messages delivered by a DCS should be strong enough to stop PWUD from consuming the product, without the need for precise quantification.

A small number of limitations relating to the implementation of LC-UV in the DrugLab DCS project were identified during the cross-validation study. First, the use of LC-UV requires highly qualified personnel, specialized laboratory equipment (precision scales, chemical hoods, laboratory benches), high-quality chemical reagents (including narcotic substances), an appropriate premises, and financial support to conduct the analyses. Some of all these structural components may not be readily available in all HR settings, limiting this method’s wider application. Second, NPS are not identified by LC-UV when detected for the first time. This means that an external laboratory (NMR or LC-HRMS) is required to identify these detected substances. Having said that, once identified, these substances can be added to the LC-UV library, as we saw with the 12 detected but unidentified products in our validation study. Finally, the number of samples included in this cross-validation analysis was limited. The preliminary results for this study need to be confirmed on a much larger number of samples and over a longer time period in order to ensure there is no analytical drift. To prevent any such drift, beyond the monthly and post-maintenance calibration controls DrugLab currently performs, the DCS is also developing a proficiency test project in HR laboratories across France, which will involve exchanging drug samples twice a year, with blind analyses conducted by each participating laboratory.

The roling-out of LC-UV in the community context will improves the assessment of the consequences of quantitative drug analyses on PWUD behaviours, and foster the participation of DCS in the national monitoring of substances on the unregulated market. Thanks to the development of the LC-UV method developed by DrugLab, this HR project has now become part of the Trans European Drug Information network (TEDI) which provides regular technical and scientific exchanges on methods and drugs through different European DCS [17]. The data collected from DCS throughout the TEDI network are uploaded on a six-monthly basis to the European Union Drugs Agency (EUDA) database, and used as a statistical and visualization tool as part of pan-European surveillance of the evolution of drug use in recreational and drug consumption room contexts.

## Conclusion

This study demonstrates the feasibility and validity of implementing a community-based quantitative DCS using LC-UV through a cross-validation analysis with LC-HRMS. The LC-UV method used by DrugLab provides accurate and reliable quantitative analyses, which are crucial for HR efforts. Despite limitations, our results confirm the method’s suitability for HR settings. Overall, our results on the implementation of LC-UV in a community setting underscore the value of robust analytical techniques which can be adapted effectively to the evolving landscape of the unregulated drug market, in the wider context of promoting the safety and well-being of PWUD.

## Data Availability

No datasets were generated or analysed during the current study.
